# Portrayals of suicide and suicide attempts among LGBTQ+ individuals in Indian digital news

**DOI:** 10.1016/j.dialog.2025.100230

**Published:** 2025-07-14

**Authors:** Khuman Bhagirath Jetubhai

**Affiliations:** School of Humanities & Social Sciences, Thapar Institute of Engineering & Technology, Patiala, Punjab, India

**Keywords:** Media portrayal, News analysis, Content analysis, LGBTQ+, Suicide, India, Public health discourse, Media representation

## Abstract

This paper examines the nature of Indian digital news reporting on suicides and suicide attempts within the LGBTQ+ community. The objective is to analyse how these incidents are framed by the media by identifying the dominant narratives, demographic characteristics highlighted, and attributed causes presented in news coverage. Following PRISMA guidelines, this study identified and analysed 164 unique digital news reports published between 2001 and 2022. The analysis reveals that news coverage most frequently focuses on individuals identified as trans women or lesbians, who are in the 19–24 and 25–44 age brackets, and are reported to be in a relationship. The most common narratives constructed in these reports attribute the suicides to family non-acceptance of their relationship or identity and partner separation. Furthermore, hanging and drug/poison ingestion are the most frequently reported methods of suicide, and joint/couple suicides are a significant feature of the media coverage. These findings demonstrate that the portrayal of LGBTQ+ suicide in Indian digital media is a curated narrative, likely shaped by journalistic news values rather than being a direct reflection of population-level trends. This research provides critical insights into the construction of media discourse on a sensitive public health issue and its implications for public perception and policy.

## 1 Introduction

Despite Indian society's general intolerance of suicide, often considering it an act of weakness or betrayal [1], suicide fatalities in the nation reached an all-time high in 2021, with 164,033 reported deaths [2]. This figure is indicative of a widespread mental health crisis, which is exacerbated by significant gaps in care; the 2016 National Mental Health Survey found that 70–80 % of people with mental illness in India do not receive the necessary treatment [3]. The WHO [4] identifies LGBTQ+ people as a particularly vulnerable group facing persecution and, consequently, a higher risk of suicide. This national crisis is even more acute for India's queer community, with studies showing that rates of clinical depression for men who have sex with men (MSM) are 6–12 times higher than in the general population [5]. Similarly, Srivastava et al. [6] found that depression risk among transgender women and MSM ranged from 11 % to 58 %, compared to 5 % to 15 % in the overall population. This poor mental health is directly linked to the stigma and prejudice the community confronts daily [7], a social climate reflected in India's relatively low rankings on global metrics like the Gay Happiness Index and the Global Acceptance Index [8,9].

Despite these alarming indicators, a literature review confirms that this area is profoundly under-researched. Kaur [10] acknowledges the limited literature on LGBTQ+ health in India, a point echoed by numerous scholars. Ramesh et al. [11] highlight the paucity of data on non-binary individuals, while Safren et al. [12] note the scarcity of data on depression across different sexual identities. Existing Indian research, as observed by Soohinda et al. [13], has narrowly focused on specific populations, such as MSM and trans women in the context of HIV or sex work. Critically, even when mental health is studied, suicide itself is often overlooked. Halli et al. [14] state that there is a lack of scientifically competent research on suicide among LGBTQ+ groups in India, and Rao [15] expresses surprise at the limited information available on suicide among the hijra community. While the National Crime Records Bureau now includes “transgender” as a gender category in its reports, it provides no details regarding sexual orientation, leaving a significant data gap [2].

Several factors contribute to this scarcity of research. Soohinda et al. [13] note that identifying homosexual individuals for studies presents a significant methodological challenge. Furthermore, sexual minorities rarely seek treatment for mental health issues or disclose suicidal thoughts due to stigma [16]. Consequently, self-harm is often unreported. When a suicide is reported, families may attribute it to other causes, like academic failure or a broken heterosexual relationship, to conceal the individual's sexual identity [17,18]. This results in a near-total lack of reliable data on the number of gay men or lesbians who have died by suicide in India.

This contemporary silence contrasts sharply with historical evidence suggesting that same-sex partnerships flourished in ancient India without overt persecution [19]. However, modern Indian society often treats homosexuality as a perversion [20], a view sometimes reinforced by a medical community that can be slow to change. As Sathyanarayana Rao et al. [21] write, “Despite its scientific base, medicine is a system sanctioned by the society in which it practises” (p. 242). In such a climate, the LGBTQ+ community faces grave mental health consequences. Furthermore, this data vacuum cannot be filled by applying findings from Western contexts. Significant cultural differences, particularly the profound societal emphasis on heterosexual marriage as a “sacrosanct union” in India, create unique pressures for LGBTQ+ individuals, who are often forced into marriages against their will [22,23]. Distinct religious views on suicide and specific mental health challenges for Indian gender minorities also render the direct application of Western data methodologically unsound [14,24]. This necessitates research rooted specifically in the Indian socio-cultural context.

Into this void of official data and culturally specific academic research steps the news media, which plays a crucial role in shaping public understanding of LGBTQ+ suicide. Despite this influence, the nature of this media portrayal has been subject to little systematic analysis. This paper addresses that gap by exploring how Indian digital news outlets frame suicides and suicide attempts among LGBTQ+ individuals. Through a systematic analysis of online news reports from 2001 to 2022, this study examines the core characteristics of these media narratives. Specifically, it analyses which demographic details are highlighted, which methods of suicide are most reported, and which precipitating events or “triggers” are constructed within the news stories. By understanding which stories are told and how they are told, this research aims to map the contours of the media discourse, providing a foundation for fostering more responsible and nuanced public conversations about LGBTQ+ mental health and suicide.

## 2 Media's role in portraying suicide

The severe mental health crisis within the LGBTQ+ community provides the critical context for this study. The public's understanding of these events is shaped by the news media, a relationship governed by established guidelines, theoretical risks, and the documented realities of reporting in India.

Global best practices for reporting on suicide are well-established. The World Health Organization's (WHO) resources for media professionals are considered the international standard, emphasising the need to avoid sensationalism and explicit descriptions of methods while promoting help-seeking behaviours [25]. In India, these principles are formally adapted into the Suicide Prevention and Implementation Research Initiative (SPIRIT) guidelines, which offer actionable advice for responsible reporting in the national context [26]. The importance of these guidelines is rooted in foundational research on media effects. Phillips' [27] seminal work documented the “Werther effect,” demonstrating that prominent, suggestive media coverage can lead to a measurable increase in imitative suicides. Conversely, more recent research has identified the protective “Papageno effect,” where stories focusing on resilience and positive coping can have a preventative influence [28]. This dichotomy reveals the media's profound responsibility, as its reporting can either contribute to harm or be a tool for prevention.

However, studies consistently show a significant gap between these guidelines and media practices in India. Research from high-suicide states like Tamil Nadu and Chhattisgarh reveals a “daily diet of short and explicit” news that directly contravenes WHO recommendations [29,30]. Crucially, this reporting is often not representative of reality, presenting a skewed picture to the public that may lead to significant misunderstandings about suicide [31]. This gap is explained by several factors. The economic pressures of journalism lead to a focus on sensationalism, a conflict captured in the title of Armstrong's research: “‘It's a battle for eyeballs and suicide is clickbait’” [32]. Furthermore, journalists themselves may hold fatalistic views on suicide and express doubts about the quality of Indian helplines, making them hesitant to include prevention resources in their reports [33]. The tangible impact of such media phenomena was highlighted by the increase in suicide-related internet searches in India following the death of celebrity Sushant Singh Rajput [34].

This problem is exacerbated when reporting on marginalised communities. For example, research in Canada shows that while traditional media can validate identity, it often presents one-dimensional and stereotypical portrayals of LGBTQ+ people [35]. Similarly, analysis of news media in Singapore reveals asymmetrical coverage of different LGBTQ+ identities, with unique and often sensationalised topics associated with each group [36]. Most pertinently to this study, research by Kar et al. [37] concludes that Indian media reporting specifically on LGBTQ+ suicide is “poorly adherent to reporting guidelines,” with frequent breaches of standards and little focus on public education.

This results in a dangerous intersection where the known risks of poor suicide reporting collide with the known problems of stereotypical LGBTQ+ representation. In the absence of official data, these flawed media narratives become the primary source of public information, making their analysis a critical imperative.

## 3 Methodology

This study was designed to systematically analyse media reports and followed the PRISMA 2020 guidelines where applicable for reporting standards [38].

### 3.1 Data sources and eligibility criteria

The primary data consisted of Hindi and English web-based news reports published between 2001 (when widespread online news archiving began in India) and 2022. The search for these reports was conducted in 2023. News reports were selected for analysis based on the following eligibility criteria:

1. The report covered either a death by suicide or a suicide attempt. Attempts were included as they are critical indicators of psychological distress and significant predictors of future death by suicide [39].
2. The report explicitly linked the suicidal behaviour to harassment, discrimination, or violence stemming from the individual's perceived or actual gender identity, sexual orientation, or non-conforming gender expression.
3. The report was published in either Hindi or English.

### 3.2 Search strategy

A comprehensive list of Indian news websites was first compiled from Wikipedia's “List of newspapers in India” [40] and supplemented with targeted Google searches. The search strategy then employed two methods: internal searches on each news website and broader searches on Google, Bing, and Yahoo. The English search terms included: ‘same-sex suicide’, ‘LGBT suicide’, ‘gay suicide’, ‘lesbian suicide’, ‘transgender suicide’, ‘bisexual suicide’, ‘queer suicide’, ‘intersex suicide’, ‘hijra suicide’, and ‘kinnar suicide’. Hindi search terms, combined with synonyms for suicide (‘जान देदी’, ‘खुदकुशी’, ‘आत्महत्या’), included: ‘बाईसेक्सुअल’ (bisexual), ‘गे’ (gay), ‘किन्नर’ (kinnar), ‘समलैंगिक’ (homosexual), ‘ट्रांसजेंडर’ (transgender), and ‘हिजड़ा’ (hijra).

### 3.3 Data selection, extraction, and analysis

The author reviewed all search results, reading each report in full to determine if it met the eligibility criteria. This screening and data extraction process was conducted solely by the author. For each of the 164 qualifying reports, the following data items were extracted into a Microsoft Excel spreadsheet:

● Year the incident was reported to have occurred
● State where the incident was reported
● Reported age of the individual(s)
● Method of suicide as described in the report
● Reported sexual/gender identity of the individual(s)
● The primary trigger event, as framed in the report's headline or lead
● Whether the report described a joint/couple or individual act
● Whether the act was a death by suicide or a suicide attempt
● The reported relationship status of the individual(s)

For consistency, this research used the umbrella term “transgender woman” to encompass indigenous South Asian gender identities (hijra, kinnar, etc.), aligning with prior research [14]. Descriptive statistics (frequencies and percentages) were generated using Microsoft 365 Excel to characterise the content of the news reports. No inferential statistical analyses were performed due to the nature of the data and the study's exploratory focus on media portrayal.

### 3.4 Limitations

The findings of this study are subject to several important limitations. First, reporting bias is inherent in the use of media data. News outlets may selectively report on cases deemed more “newsworthy,” potentially over-representing sensational stories and under-representing others. Furthermore, due to social stigma, the LGBTQ+ identity of an individual may be concealed or misreported in the news. Second, the data is limited by the information available in the reports, which often lack crucial context about an individual's background or mental health history. Third, the single-author approach to data selection and extraction introduces potential selection bias based on the author's interpretation of the reports. Finally, the certainty of the evidence is considered low due to the reliance on secondary news sources rather than primary data, the lack of a comparison group, and the study's retrospective nature. Consequently, the findings of this study should not be interpreted as reflecting the epidemiological reality of suicide in India. Instead, they offer a systematic mapping of the media discourse surrounding LGBTQ+ suicide, providing exploratory insights into which narratives are prioritised and how these sensitive events are framed for public consumption.

## 4 Result

This section presents the findings from the content analysis of the 164 qualifying digital news reports, which revealed distinct patterns in how LGBTQ+ suicides and suicide attempts are portrayed in Indian digital news. Of the incidents covered, 88 % were described as deaths by suicide and 12 % as attempted suicides, a distribution similar to the findings of Kar et al. [37]. The key characteristics of the media coverage are detailed in Table 1.

**Table 1:** Characteristics of Indian digital news reports on LGBTQ+ suicide and suicide attempts (*N* = 164).

Reporting Year	Number	Reported State	Number	Reported Age	Number	Reported Method of Suicide	Number
2004	2 (1 %)	Tamil Nadu	31 (19 %)	Adolescent (13–18)	31 (19 %)	Hanging	67 (41 %)
2005	2 (1 %)	Madhya Pradesh	19 (12 %)	Young Adult (19–24)	59 (36 %)	Poison/drug indigestion	39 (24 %)
2006	3 (2 %)	Uttar Pradesh	19 (12 %)	Adult (25–44)	52 (32 %)	Burn	16 (10 %)
2007	2 (1 %)	Maharashtra	19 (12 %)	Middle-aged (45–64)	3 (2 %)	Being run over by a train	14 (9 %)
2008	7 (4 %)	West Bengal	15 (9 %)	Aged (65–120)	1 (1 %)	Fall from height	7 (4 %)
2009	2 (1 %)	Gujarat	11 (7 %)	Not mentioned	18 (11 %)	Drowning	8 (5 %)
2010	3 (2 %)	Kerala	9 (5 %)	Average age	18	Cut/pierce	5 (3 %)
2011	6 (4 %)	Jharkhand	8 (5 %)	Reported Sexual/Gender Identity	Number	Not mentioned	8 (5 %)
2012	11 (7 %)	Bihar	5 (3 %)	Transwoman	56 (34 %)	Reported Trigger Event	Number
2013	5 (3 %)	Chattisgarh	5 (3 %)	Lesbian	53 (32 %)	Family unacceptance of love/identity	77 (47 %)
2014	8 (5 %)	Haryana	4 (2 %)	Gay	35 (21 %)	Breakup/separation	21 (13 %)
2015	6 (4 %)	Odisha	4 (2 %)	Trans attracted man	7 (4 %)	Unclear	17 (10 %)
2016	10 (6 %)	Karnataka	4 (2 %)	Gender nonconforming	6 (4 %)	Institutional discrimination⁎	15 (9 %)
2017	6 (4 %)	Telangana	3 (2 %)	Transman	4 (2 %)	Non-institutional discrimination⁎	14 (9 %)
2018	18 (11 %)	Assam	2 (1 %)	Intersex	1 (1 %)	Chronic illness	7 (4 %)
2019	22 (13 %)	Andhra Pradesh	1 (1 %)	Bisexual woman	1 (1 %)	Argument	5 (3 %)
2020	21 (13 %)	Jammu Kashmir	1 (1 %)	Crossdresser	1 (1 %)	Guilt	4 (2 %)
2021	15 (9 %)	Delhi	1 (1 %)	Death by Suicide or Suicide Attempt	Number	Financial issues	2 (1 %)
2022	15 (9 %)	Manipur	1 (1 %)	Died by suicide	144 (88 %)	Superstitious beliefs	1 (1 %)
Joint/couple or Individual suicide	Number	Punjab	1 (1 %)	Attempted suicide	20 (12 %)	Other trigger events	1 (1 %)
Single	97 (59 %)	Puducherry	1 (1 %)	Reported Relationship status	Number		
Joint	67 (41 %)			In relationship	92 (56 %)		
				Single	19 (12 %)		
				Unclear	53 (32 %)		

⁎ Non-institutional discrimination includes discriminatory behaviour by neighbours, friends, acquaintances, onlookers, and the general public. Institutional discrimination, on the other hand, refers to discriminatory practices, policies, or procedures within schools, workplaces, organisations, or other formal institutions.

The analysis of the news report corpus showed that the volume of reporting on this topic peaked in 2019 and 2020 (13 % each), with another significant increase in 2018 (11 %). Geographically, the coverage was concentrated, with the highest number of reports originating from Tamil Nadu (19 %). Notably, despite their large populations, no reports from Rajasthan, Himachal Pradesh, or Uttarakhand were identified in the dataset, a finding that likely reflects regional differences in media coverage rather than an absence of such events. The news reports tended to focus on specific demographic profiles, with young adults (19–24 years) and adults (25–44 years) being the most frequently portrayed age groups, at 36 % and 32 %, respectively. In contrast, there was minimal media coverage of middle-aged (2 %) and older adults (1 %). In terms of reported identity, the individuals in the news reports were most frequently identified as trans women (34 %), lesbians (32 %), and gay men (21 %). This distribution is similar to the findings of Kar et al. [37], who also noted a high representation of trans women, lesbians, and gay men in their analysis of Indian newspaper reports. A majority of the reports (56 %) described individuals who were in a relationship.

The news reports also constructed clear narratives around the methods and triggers of suicide. Hanging was the most frequently described method of suicide (41 %), followed by poison or drug ingestion (24 %). This focus is consistent with reporting on broader suicide trends in India. While hanging was also the most common method in the study by Kar et al. [37] at 35 %, they found drowning to be the second most frequent at 21 %, whereas in this study's corpus, drowning accounted for only 5 % of reported methods. The most common narrative trigger constructed in the reports was family non-acceptance of the individual's identity or relationship (47 %), followed by partner separation or breakup (13 %). Finally, a significant portion of the reports (41 %) covered joint or couple suicides. The prominence of this narrative in the media corpus reflects a phenomenon previously observed in research on lesbians in India by Vanita [41].

## 5 Discussion

### 5.1 The portrayal of age in suicide reporting

As the results demonstrate, the news reports in this study's corpus predominantly featured adolescents (13–18), young adults (19–24), and adults (25–44), with minimal coverage of middle-aged and older individuals. This pattern of media focus is consistent with established literature on reporting biases and news values. The intense focus on younger individuals can be interpreted through the lens of “newsworthiness.” Research by Armstrong et al. [32] found that media professionals in India explicitly ascribe greater newsworthiness to the suicides of youths and students. This suggests that the age distribution found in this study's data may directly reflect a journalistic tendency to prioritise stories about the young. Conversely, the lack of coverage for older adults aligns with the observation by De Leo [42] that suicides in this demographic are particularly prone to under-reporting. De Leo suggests that the advanced age of the deceased may lead to less investigative interest compared to a death in youth, or the death may be attributed to other factors like medical complications. Therefore, the age distribution observed in the analysed news reports likely reflects these powerful journalistic norms, where the perceived newsworthiness of youth suicide contrasts with the relative invisibility of suicide among older adults in media discourse.

### 5.2 The portrayal of suicide methods in news reports

The analysis of the media corpus showed that hanging and poisoning were the most frequently described methods of suicide. However, the prevalence of specific methods in news reports may be more indicative of media selection bias than their actual frequency in the population. Research on journalistic practices confirms that the perceived “newsworthiness” of a suicide method heavily influences its likelihood of being reported. For instance, Armstrong et al. [31] found that Indian media tend to over-represent methods with high lethality and public spectacle, such as hanging or jumping, while under-representing others. The finding that hanging is the most frequently reported method in this study's corpus aligns with this demonstrated media bias. This selective focus is further highlighted by situational reporting trends; for example, Pathare [43] noted an increase in the reporting of hanging during the COVID-19 lockdown. Ultimately, these studies show that the methods of suicide that become prominent in the public discourse are shaped by a complex set of reporting biases and are not necessarily a direct reflection of their prevalence within the community.

### 5.3 Dominant identity portrayals in suicide news reports

A significant finding from this study is the dominant media portrayal of specific queer identities within suicide news reports. The analysis revealed a disproportionately high number of cases involving trans women and lesbians, a finding consistent with other Indian media analyses where transgender people (44.4 %) and lesbians (27.4 %) were also the most frequently represented groups in suicide reports [37]. The notable absence of other identities within the LGBTQ+ spectrum in the reports aligns closely with established patterns of media erasure. For instance, research confirms that bisexuality is significantly under-represented in media [44]. This is likely compounded in suicide reporting, where individuals in outwardly heterosexual relationships would not be identified as bisexual unless explicitly stated. Similarly, asexuality is rendered almost non-existent in mainstream narratives, leading to its status as the “invisible orientation” and explaining its absence from our data [45,46]. A parallel lack of representation exists for trans-masculine individuals in media and screen cultures, further accounting for their invisibility in the collected reports [47].

The heightened visibility of trans women and lesbians, in contrast, may be explained by their perceived alignment with specific news values, though this visibility is often problematic. As Sukthankar [48] observed, the history of lesbian visibility in India has been like “scattered fireworks,” where isolated episodes are “digested with prurient relish and then forgotten,” leaving no trace of the actual lives lived [48]. This results in a media landscape that focuses on the “cataclysmic aspects” of lesbian lives, such as couple suicides, rather than nuanced representation [48]. This historical pattern of sensationalism helps explain why narratives centred on individuals perceived by society as more vulnerable—a perception often applied to women and transgender people—can be framed to generate stronger emotional responses from an audience. Furthermore, stories involving transgender individuals may be prioritised by some outlets as they offer opportunities to include details often framed as sensational or novel. This can involve a focus on medical transitions or the construction of a victimhood narrative centred on experiences of violence, discrimination, poverty, or sex work, all of which can increase a story's perceived newsworthiness. Therefore, the skewed representation of identities in the media corpus appears to be a dual phenomenon: the systemic erasure of less visible identities combined with a journalistic preference for sensational narratives.

### 5.4 Portrayal of suicide triggers

The finding that family non-acceptance (47 %) and relationship breakups (13 %) were the most frequent triggers portrayed in the news reports warrants significant discussion. The prominence of these narratives suggests that news reports about LGBTQ+ suicide may be selected for publication based on their alignment with established “news values”. Harcup and O'Neill [49] identify factors such as conflict, drama, surprise, and tragedy as key elements that make a story newsworthy. Narratives focused on family rejection or romantic loss within a non-heteronormative context fit these criteria perfectly. They provide clear elements of interpersonal conflict (individual vs family) and drama, creating a compelling story for the public. The focus on identity as the reason for family rejection—rather than a crime or a specific action—adds a layer of tragedy that can be framed as particularly shocking to a general audience. This may explain why these emotionally charged narratives are more prevalent in the media corpus than other, perhaps more complex or less “dramatic,” contributing factors to suicide. Furthermore, the prevalence of these specific narratives may help explain the demographic focus on youth in the media coverage. The life stages of adolescence and young adulthood are culturally understood as key periods for identity formation, first romantic relationships, and the process of “coming out” to family. Therefore, media narratives that frame suicide around the themes of family rejection and romantic loss inherently align with the portrayal of younger individuals. This suggests the overrepresentation of these triggers in the news may reflect not only their tragic reality but also their high degree of newsworthiness, which prioritises stories with strong narrative and emotional components.

### 5.5 The over-representation of suicide pacts

A notable finding from this study is the high prevalence of joint or couple suicides, which constituted 41 % of the analysed news reports. This high proportion likely reflects media selection bias rather than the actual frequency of such events. The intersection of two statistically rare phenomena—suicide pacts and LGBTQ+ identity—appears to create an event of exceptional perceived newsworthiness. This aligns with the findings of Sharma et al. [50], who note that rare occurrences like suicide pacts receive disproportionate media attention, likely because news professionals deem them more sensational than more common suicide incidents. Furthermore, these joint suicide reports often conform to powerful, pre-existing cultural narratives of tragic, “star-crossed” lovers who face insurmountable societal opposition. By framing the event within this familiar archetype, the story gains a dramatic structure that is highly compelling. The prominence of this tragic romance narrative may also explain this study's finding that lesbians were one of the most frequently reported demographics, as many of these joint suicide stories involved female couples. Thus, the over-representation of suicide pacts in the media corpus appears to be a clear example of journalistic selection favouring stories that are rare, sensational, and align with established dramatic storytelling conventions.

## 6 Conclusion

This analysis of Indian news reports on LGBTQ+ suicides from 2001 to 2022 concludes that media portrayal is heavily skewed by perceived “newsworthiness” rather than a factual representation of the crisis. This media bias is evident across all findings. The disproportionate focus on sensational elements such as joint suicides or suicide pacts, alongside fatal methods like hanging and poison ingestion, highlights this trend. Similarly, the data suggests a preference for stories with higher shock value, evident in the over-representation of younger individuals (19–44 years), while the deaths of older adults (45–120) are largely ignored. Thematic news values also appear to drive the narrative, with family non-acceptance and relationship breakups emerging as the most prominent triggers. Furthermore, this reporting privileges certain identities—with a high representation of gay men, trans women, and lesbian women—while rendering other identities, like bisexual individuals and trans men, nearly invisible. Ultimately, this study suggests that the public narrative of LGBTQ+ suicide in India is a media construct, shaped more by the demands of sensationalism and reader interest than by the complex realities of the queer community's mental health epidemic.

## 7 Recommendations

This study's findings indicate that news coverage of LGBTQ+ suicide in India frequently focuses on tragic and dramatic narratives. While this reporting highlights a real crisis, it carries the significant risk of normalising suicide within public perception. The danger of such repeated, tragic media portrayals is that they can contribute to a fatalistic belief that suicide is an inevitable part of the LGBTQ+ life trajectory. This concern is captured by M. Rao [15], who warns against viewing these tragedies “as though this is how they are meant to live, haunted by suicide” (p. 207). When the media inadvertently frames suicide as a “normal” outcome for a specific community, it can undermine the urgency of preventative action. This perception may lead to institutional apathy, where the underlying factors of discrimination and violence are not taken seriously by authorities [51]. Therefore, a primary recommendation is for public health advocates, community leaders, and media watchdogs to actively challenge media narratives that frame LGBTQ+ suicide as unavoidable. Furthermore, media organisations can proactively counter this narrative. A crucial recommendation is for news outlets to balance tragic stories with portrayals of hope and resilience. As research by Niederkrotenthaler et al. [52] demonstrates, media stories focusing on recovery from suicidal crises can have a beneficial effect on vulnerable individuals. Intentionally including these positive narratives—an approach known as the Papageno effect—is an evidence-based, preventative strategy that the media can adopt. Additionally, a key recommendation is developing and implementing specialised training for journalists. Reporting on LGBTQ+ suicide lies at the intersection of two complex fields—trauma reporting and LGBTQ+ community issues, both of which require awareness and skills that standard journalism courses may not provide. One crucial component of this training should be “trauma-informed journalism” [53]. This approach moves beyond simply reporting facts to understanding the profound impact of traumatic events on survivors. As Mark Brayne [54] observes, journalists have a “profound responsibility to tell the story well,” as their work can either calm or “exacerbate—the grief and distress that ripples out from death and injury”. This means understanding how a journalist's actions will impact a survivor long after the story is published [qtd. in 53]. In parallel, sensitive reporting on this topic requires specific training on LGBTQ+ issues. According to ILGA-Europe [55], when journalists are well-informed, they can “make conscious approaches to news affecting LGBTI people and draw sensitive depictions of LGBTI subjects” (p. 1). By combining trauma-informed practices with specific LGBTQ+ awareness, media organisations can foster a more ethical, nuanced, and responsible form of reporting that minimises harm and better serves the public.

## 8 Limitations and further research

This study has several limitations that should be acknowledged. First, the analysis relies on the identity labels (e.g., ‘lesbian,’ ‘trans woman’) used within the news reports themselves. These media-assigned labels may oversimplify the fluid and complex nature of an individual's self-identity, particularly for the younger individuals who were frequently the subject of these reports [56]. Second, the focus on a single “trigger event” is a limitation imposed by the nature of news reporting. Suicide is a multifactorial phenomenon with numerous interacting determinants [37]. News narratives, however, often prioritise a single, recent event over the complex history of an individual's experiences [57]. Therefore, this study's analysis of reported triggers reflects the media's narrative simplification rather than the complete circumstances of the suicidal act. Finally, a methodological limitation is the single-author approach to data screening and extraction. Although this was necessitated by resource constraints, the study lacks the cross-validation provided by multiple reviewers, which is recommended by PRISMA guidelines to minimise bias and enhance reliability [38].

Future research could conduct comparative content analyses of LGBTQ+ suicide reporting across different media platforms (e.g., print, television, social media) to identify variations in framing, sensationalism, and adherence to ethical guidelines. Furthermore, given the relative invisibility of bisexual and trans men in this study's media corpus, dedicated research is urgently needed. This should include not only studies on the specific mental health challenges faced by these groups but also media studies exploring the reasons for their under-representation in public discourse. Finally, audience reception studies are needed to assess how these portrayals impact the perceptions and attitudes of both the general public and members of the LGBTQ+ community themselves.

## Author contribution

The author confirms sole responsibility for the following: study conception and design, data collection, analysis and interpretation of results, and manuscript preparation.

## Ethics statements

This article does not contain any research that required ethical approval or oversight, as no human or animal subjects were involved.

## Declaration of generative AI and AI-assisted technologies in the writing process

During the preparation of this work the author(s) used ChatGPT in order to proofread the article. After using this tool/service, the author(s) reviewed and edited the content as needed and take(s) full responsibility for the content of the publication.

## Funding information

No funding was received for conducting this study. The authors have no relevant financial or non-financial interests to disclose.

## Declaration of competing interest

The authors certify that they have NO affiliations with or involvement in any organization or entity with any financial interest (such as honoraria; educational grants; participation in speakers' bureaus; membership, employment, consultancies, stock ownership, or other equity interest; and expert testimony or patent-licensing arrangements), or non-financial interest (such as personal or professional relationships, affiliations, knowledge or beliefs) in the subject matter or materials discussed in this manuscript.

## Data Availability

The data underlying the results presented in the study are available at https://osf.io/mzqb2/?view_only=5947de1c74cd4fe1a5c2f8d09bf72583
